# A CONSORT-compliant prospective randomized controlled trial: radiation dose reducing in computed tomography using an additional lateral scout view combined with automatic tube current modulation: Phantom and patient study

**DOI:** 10.1097/MD.0000000000007324

**Published:** 2017-07-28

**Authors:** Wanlin Peng, Zhenlin Li, Chunchao Xia, Yingkun Guo, Jinge Zhang, Kai Zhang, Lei Li, Fei Zhao

**Affiliations:** a Department of Radiology, West China Hospital of Sichuan University, Chengdu; b Department of Radiology, Key Laboratory of Obstetric & Gynecologic and Pediatric Diseases and Birth Defects of Ministry of Education, West China Second University Hospital, Chengdu, China.

**Keywords:** computed tomography, quality control, radiation dose, thorax

## Abstract

**Background:**

Radiation exposure has been a hot point in research field of computed tomography (CT). Recently, automated tube current modulation (ATCM) has emerged as an important technique to reduce radiation exposure. Many studies have shown that the difference in scout view would affect modulation. This prospective randomized controlled study is aimed to investigate the impact of an additional lateral scout view on radiation dose and image quality in CT using ACTM.

**Methods:**

Combined with ATCM (Care Dose 4D) on multidetector CT, 2 thoracic phantom CT image series were acquired in which planning was conducted with either an anteroposterior (AP) or an AP-lateral scout view. Also, 410 patients underwent thoracic CT examinations using Care Dose 4D modulation and were randomized to either a scan planned with an AP-lateral scout or a single AP scout. Effects of the different scout views on applied effective milliampere seconds (mAs), volume CT dose index (CTDIvol) and dose–length–product (DLP) were analyzed. The quality of patient CT images was also assessed. Data were analyzed using independent *t* tests and linear correlation analysis.

**Results:**

Compared with AP groups, the mean CTDIvol (phantom, 0.89 ± 0.08 vs 1.36 ± 0.26 mGy, *P* < .001; in patients, 1.12 [0.96, 1.34] vs 2.16 [1.66, 2.64] mGy, *P* < .001) and DLP (in phantom, 26 [23.25, 28] vs 40 [34.25, 48] mGy×cm, *P* < .001; in patients, 41 [33, 41] vs 77 [60.5, 99.5] mGy×cm, *P* < .001) were significantly reduced by approximately 50% in AP-lateral scout view group. With the AP-lateral topogram, the radiation dose on different off-center positions was essentially equal (CTDIvol: 0.76–0.99 mGy; DLP: 22–28 mGy×cm effective dose: 0.31–0. 39 mSv). For image quality, contrast-to-noise ratio and signal-to-noise ratio values in the AP group were similar to those of AP-lateral scout view group.

**Conclusion:**

AP combined with an additional lateral scout view using ACTM can significantly reduce the radiation dose without compromising image quality in chest screening CT.

## 1 Introduction

The use of computed tomography (CT) has increased greatly in the past few years.^[1,2]^ Medical imaging accounts for 48% of the radiation exposure in the United States, with half of all medical radiation exposure resulting from CT.^[3]^ Furthermore, the use of CT as a screening tool also exposes large numbers of asymptomatic individuals to repeated radiation exposure.^[4–6]^ Exposure to ionizing radiation, even at the relatively low doses used in screening, is associated with incrementally increased risks of cancer, especially in women and young people.^[3,7]^ Radiation exposure from CT scans at a young age may increase the risk for tumors and leukemia in later life.^[8,9]^ To avoid unnecessary radiation exposure, scanning techniques should be optimized to always conform to the “as low as reasonably achievable” (ALARA) principle proposed by the International Commission on Radiological Protection.^[10]^

Various strategies have been adopted to reduce radiation exposure during CT imaging, including increasing the pitch,^[11]^ lowering the tube potential,^[12]^*z* axis automated tube current modulation (ATCM),^[13]^ and using iterative reconstruction algorithms.^[14]^ ATCM techniques have simplified the task of scanning parameter individualization compared with the aforementioned methods so that the radiation dose can be minimized without deteriorating image quality. Tube current is varied on the basis of a scout view and real-time feedback of actual attenuation during rotation. The type of scout views used have a great impact on the level of radiation exposure with ATCM.^[15–19]^ The manufacturer (Siemens Healthcare, Germany) indicates that ATCM uses information of all valid topograms to calculate milliampere seconds values. A previous report has suggested using lateral scout views for planning CT to reduce radiation dose,^[17]^ but attenuation information in the anteroposterior (AP) direction is merely an estimate and inaccurate. A recent study using cadavers has shown that using 2 orthogonal scout views (AP and lateral) for ATCM could reduce radiation dose more than a single AP scout view.^[15]^ To our knowledge, no studies have evaluated the clinical utility of an additional lateral scout view or its impact on image quality. Thus, we conducted a prospective, randomized controlled trial to compare the effect of AP + lateral to programs to single-view AP to programs on radiation dose and image quality using ATCM.

## 2 Material and methods

### 2.1 Study protocol and patients data

This randomized controlled clinical study was designed according to the CONSORT 2010 statement.^[20]^ This study was approved by the Institutional Medical Ethics Committee of West China Hospital of Sichuan University (No. 2014-163), and written informed consents, which included enduring the level and reaction of radiation exposure, were obtained from all patients prior to the CT examination. No intervention was provided to participants, with strict secrecy for personal information and privacy.

From February 2016 to May 2016, we consecutively enrolled healthy patients who underwent a routine thoracic CT examination at our hospital. Exclusion criteria included inability to cooperate with the examination, age younger than 18 years, renal dysfunction (estimated glomerular filtration rate < 60 mL/min/1.73 m^2^) or severe left ventricular dysfunction (ejection fraction < 35%), severe motion artifacts or metal artifacts, and body mass index (BMI) >30 kg/m^2^ (patients were classified as obese if their BMI >30 kg/m^2^).^[21]^ According to the data obtained from a pilot study involving 40 subjects, we planned to recruit at least 207 subjects into our study. The subjects were randomly assigned either to a test group that received an AP and an additional lateral scout view or to a control group that received only an AP scout view. For a self-control study, we retrospectively extracted last thoracic CT examinations with an AP scout view of patients in test group, if they have. The change of body weight for which included in self-control study was <2 kg between the 2 examinations.

### 2.2 Phantom CT scanning

Phantom images were acquired on a 128-slice multidetector CT scanner (Siemens Somatom Definition AS+; Siemens Healthcare) with 64 detector rows and a *z*-flying focal spot. An anthropomorphic phantom (CPD-R2; Chengdu Phantom Emulation Technology Co., Ltd., Chengdu, Sichuan, China) was scanned at 20 different vertical patient table positions. The isocenter position was visually set by the radiographer. Table height was varied from 10 cm below to 10 cm above the isocenter position in 1-cm increments, although the highest position of the patient table was set at 9.5 cm due to limits to the vertical movement of the table.

**Figure 1:**
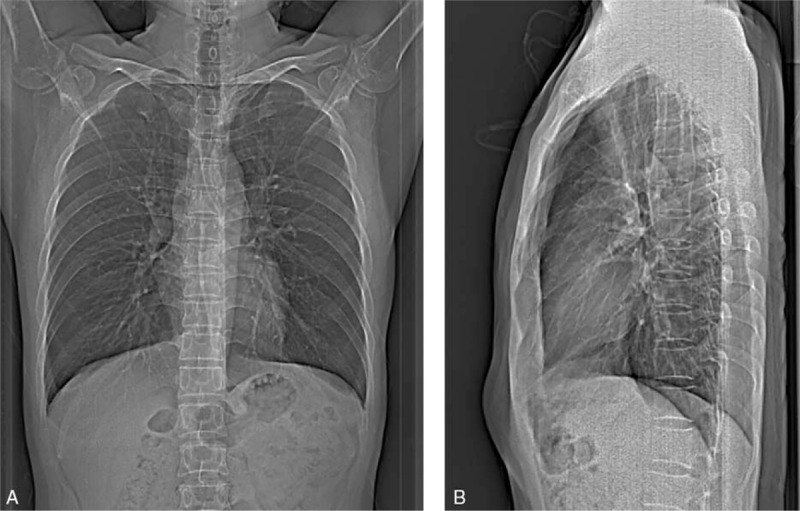
An example of the AP (A) and lateral (B) scout views used for chest CT scanning. AP = anteroposterior, CT = computed tomography.

To obtain the AP and lateral scout views (Fig. 1), chest images were acquired at 80 kV and 35 mA. The scan length was set to that of a typical chest CT examination (from the apices of the lungs to the lateral phrenic angles). Specific imaging parameters were as follows: 1.2:1 pitch, 128 × 0.6 mm detector configuration, 0.6 mm beam collimation, 61.4 mm/rotation table speed, 0.5 s gantry rotation time, 5-mm reconstructed section thickness with 5-mm reconstruction increment, 120 kV tube voltage, ATCM with a quality reference tube–current–time product of 30 mAs and ACTM by Care Dose 4D with an “average” modulation strength.

### 2.3 Patient CT examination

All patients were scanned in the supine position in the craniocaudal direction with arms raised above the head. An automated verbal command instructed all patients to take a breath and hold it during imaging. Scanning parameters coincided with those used in the phantom study.

### 2.4 Radiation exposure

Applied effective milliampere seconds, which had been automatically adjusted by ATCM based on the defined reference tube current–time products, volume CT dose index (CTDIvol) and dose–length–product (DLP), were recorded for each CT scan. Then effective dose (ED) was calculated from the DLP multiplying by a dose conversion factor of 0.014 mSv/mGy.^[22]^ Body weight (kg) and height (m) of all patients were recorded during scanning to calculate BMI. The scan length, which has a direct impact on the DLP, was also recorded for all patients.

### 2.5 Image quality assessment

**Figure 2:**
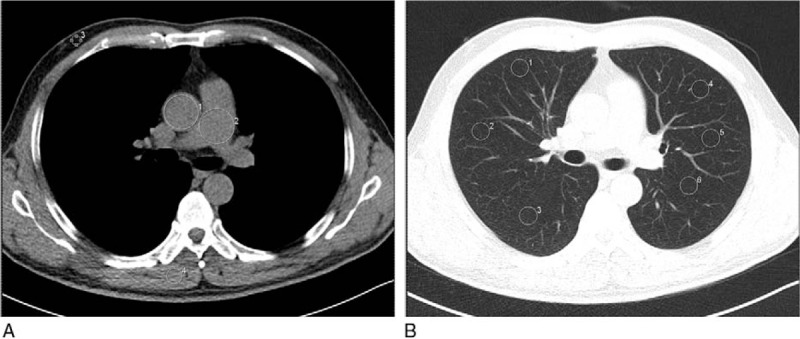
An example of ROI of measurements on transverse unenhanced multidetector CT images. For all measurements, the size, shape, and position of the ROIs were kept approximately constant across patients. (A) ROIs manually drawn on the descending aorta (ROI 1), pulmonary trunk (ROI 2), subcutaneous fat of the anterior abdominal wall (ROI 3), paraspinal muscle (ROI 4) and air (ROI 5); (B) ROIs manually drawn on the pulmonary parenchyma (ROIs 1–6). CT = computed tomography, ROI = range of interest.

To obtain mean CT attenuation values, we manually drew a circular region of interest (ROI) of approximately 150 mm^2^ in the ascending aorta, the pulmonary trunk, the paraspinal musculature and the lung parenchyma at the level of the carina (Fig. 2). Calcifications and soft plaques on the aortic wall were avoided as much as possible. Subcutaneous fat of the prothorax wall was recorded from a single ROI of approximately 50 mm^2^. Noise was measured in a circular ROI of 50 mm^2^ placed in an artifact-free region (air) 3 cm ventral of the thoracic wall.

For each participant, the signal-to-noise ratios (SNRs) and contrast-to-noise ratios (CNRs) were calculated by the equations: SNR = ROI_o_/SD_air_ and CNR_o-fat_ = (ROI_o_ − ROI_fat_)/SD_fat_, where ROI_o_ is the mean attenuation for the organ of interest, SD_air_ is the standard deviation (SD) for artifact-free region (air), ROI_fat_ is the mean attenuation for the subcutaneous fat of the anterior abdominal wall, and SD_fat_ is the SD.^[17]^

Images were displayed with a lung window setting of window width (WW) = 1200 hounsfield unit (HU), window level (WL) = −600 HU and a mediastinal window setting of WW = 400 HU, WL = 40 HU. The observers were allowed to modify settings depending on the actual conditions to obtain more distinct images. All images were assessed for clarity of visualization of the bronchi and vessels in lung images, as well as the mediastinum, aorta, lung vasculature, and chest wall in mediastinum images. Subjective image quality was quantitatively assessed using a 5-point system^[23]^: 5, excellent, no blurring or artifact-interfered diagnosis; 4, good, slight blurring or noise with unrestricted image evaluation possible; 3, moderate, moderate blurring or noise with slightly restricted image evaluation; 2, poor, diagnostic confidence significantly reduced but can make a diagnosis; and 1, unacceptable, indistinct delineation of vessel and bronchial margins, or excessive noise with no diagnosis possible.

### 2.6 Reproducibility assessment

Two thoracic radiologists with >3 years of experience who were blinded to patient data and group assignment independently assessed the transverse images of all patients for image quality. Each radiologist was asked to perform a repeat analysis after 3 weeks to provide intraobserver reliability data.

### 2.7 Statistical analysis

A D’Agostino–Pearson test was performed to test for normal distribution of data.^[24]^ Normally distributed continuous data were expressed as mean ±  SD. Conversely, nonnormally distributed data were expressed as median (interquartile range, IQR) according to the normal distribution test of data, the between-group differences in mean radiation dose delivered to patients in the test and the control groups was analyzed using the independent samples *t* test or Mann–Whitney *U* test. The differences between the scores of image quality was analyzed using the Wilcoxon rank-sum test. For the same patient, within-patient differences in mean radiation dose between the 2 CT examinations was analyzed using the paired *t* test. Pearson correlation was performed to determine the relationship between BMI and CTDIvol for the AP and AP-lateral topogram groups. Intra- and interobserver agreements were analyzed using the linear-weighted interrater agreement (Kappa) test with a calculation of 95% confidence interval (CI). Statistical analyses were performed using Excel 2010 (Microsoft Corp, Redmond, WA) and SPSS version, 23.0.0 (SPSS, Inc, Chicago, IL). *P* < .05 was considered to be statistically significant.

## 3 Results

### 3.1 Phantom study

**Figure 3:**
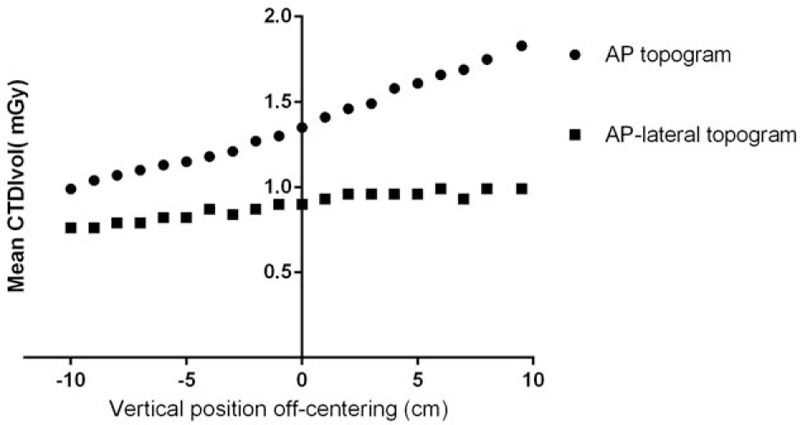
CTDIvol at different off-center positions when an AP or AP-lateral topogram was used for ATCM. When AP scout views were used for ATCM, radiation dose gradually reduce as the table height decreased and far away from x-ray tube. The radiation dose on different off-center position was essentially flat when AP-lateral topogram was used for ATCM. AP = anteroposterior, ATCM = automated tube current modulation, CTDIvol = volume CT dose index.

In the phantom study, the x-ray tube was on the top of the gantry. When the phantom was positioned in the isocenter, the radiation doses using AP and AP-lateral scout images were 0.56 (0.48, 0.67) mSv and 0.36 (0.33, 0.39) mSv, respectively. When the phantom was positioned 10 cm above to 9.5 cm below the gantry isocenter, the tube current–time product ranged from 30 to 55 mGy×cm with AP scout view and 22 to 28 mGy×cm with AP-lateral scout views. The radiation dose was essentially similar when the AP-lateral topogram was used for ATCM (Fig. 3). The applied effective milliampere seconds, CTDIvol, DLP, and ED associated with ATCM using either AP or AP-lateral topograms are shown in Table 1. There was a statistically significant difference in radiation dose associated with ATCM between the AP and AP-lateral scout views (*P* < .001).

**Table 1:**
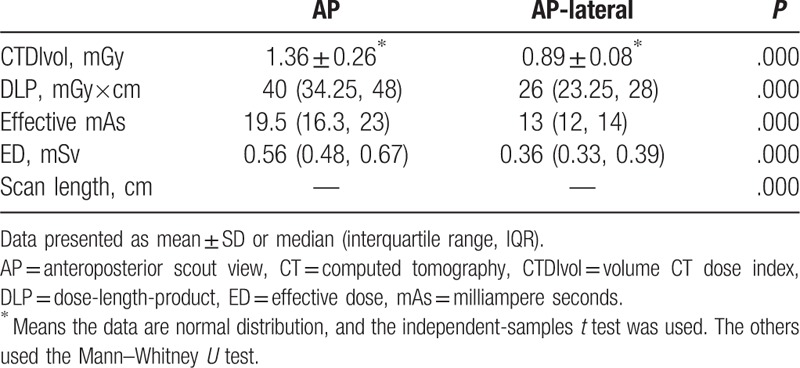
CTDIvol, DLP, mean effective mAs, ED, and scan length for CT scans of the phantom.

### 3.2 Patient study

After exclusion, 410 patients (225 men, 185 women; mean age 47.2 ± 10.54 years, range 23–82 years; mean BMI 23.84 kg/m^2^) were recruited into our study. The test group consisted of 112 men and 93 women, aged 47.42 ± 11.48 years (range 23–78 years) and a mean BMI of 23.89 kg/m^2^. The control group consisted of 111 men and 99 women, aged 46.99 ± 9.53 years (range 25–82 years), with a mean BMI of 23.79 kg/m^2^. There were no significant differences between the 2 patient groups with regard to sex, age, and BMI.

**Figure 4:**
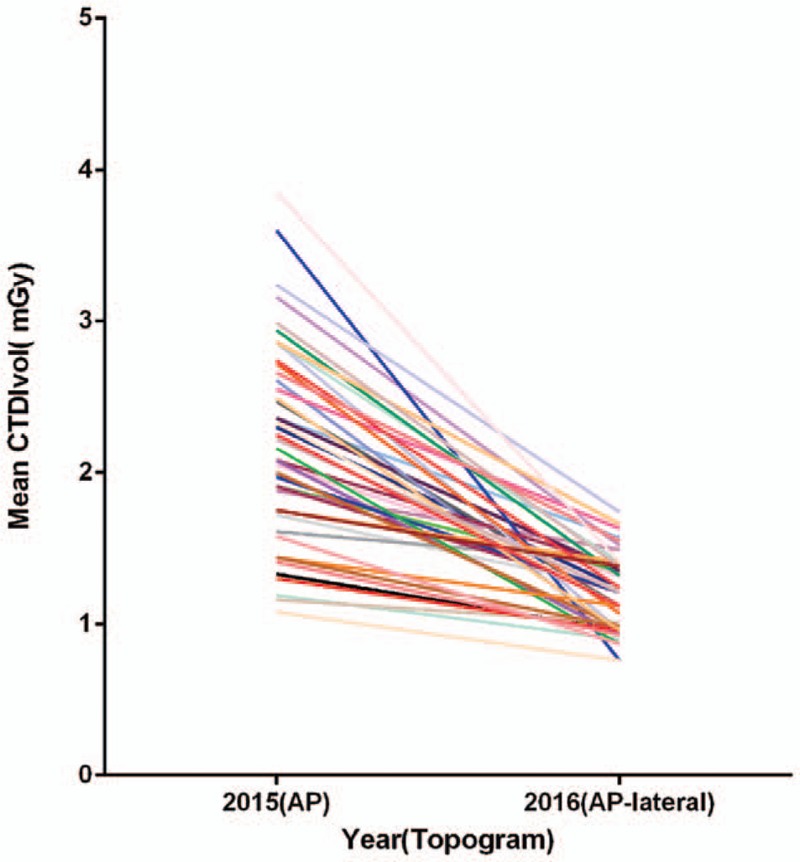
Intraindividual comparison of CTDIvol between CT scans planned on AP and AP-lateral scout views. AP = anteroposterior, CT = computed tomography, CTDIvol = volume CT dose index.

**Figure 5:**
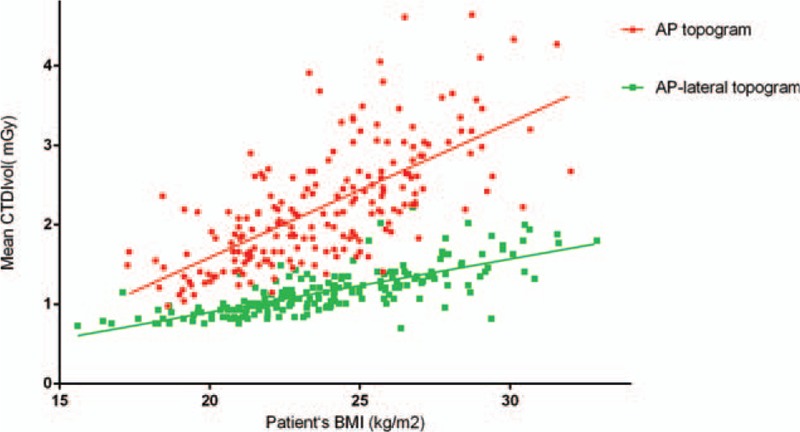
Scatter diagram showing the correlation between CTDIvol and patient BMI when AP or AP-lateral topograms were used. AP = anteroposterior, BMI = body mass index, CTDIvol = volume CT dose index.

Table 2 shows there was a substantial decrease in the mean ED with the use of an additional lateral scout view (1.08 [0.85, 1.39] vs 0.57 [0.46, 0.69] mSv; *P* < .001). With regard to CTDIvol and DLP, the use of an AP-lateral scout view resulted in a reduction in radiation dose of approximately 50% compared with the AP scout view. In the 44 patients (29 men, 15 women; mean age 50.7 ± 11.08 years, range 28–79 years) who underwent 2 thoracic CT examinations in 2 years, the results were similar to findings for the 2-group comparison (Fig. 4). There was a moderate correlation between CTDIvol and patient BMI when an AP (R^2^ = 0.48) or AP-lateral (R^2^ = 0.58) topogram was used (Fig. 5).

**Table 2:**
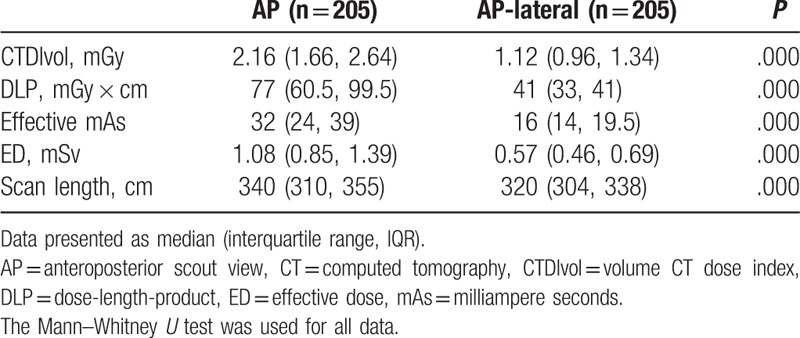
CTDIvol, DLP, mean effective mAs, ED, and scan length for CT scans of the patients.

### 3.3 Image quality assessment

As Table 3 shows, the SNR of the aorta, fat, and pulmonary parenchyma were significantly decreased when planned on an AP-lateral scout view as opposed to an AP scout view, and the SNR of the muscle was similar. A significantly lower CNR of the aorta relative to fat (13.07 [11.16, 14.40] vs 11.18 [9.72, 12.90]) was observed in the AP-lateral group compared with the AP group.

**Table 3:**
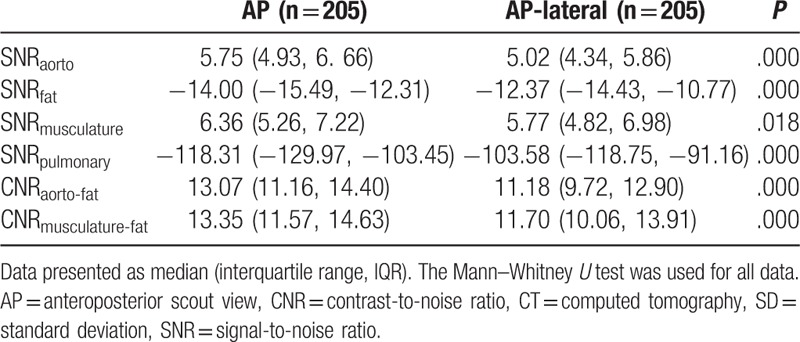
SNRs and CNRs of chest CT examinations in 410 patients.

**Figure 6:**
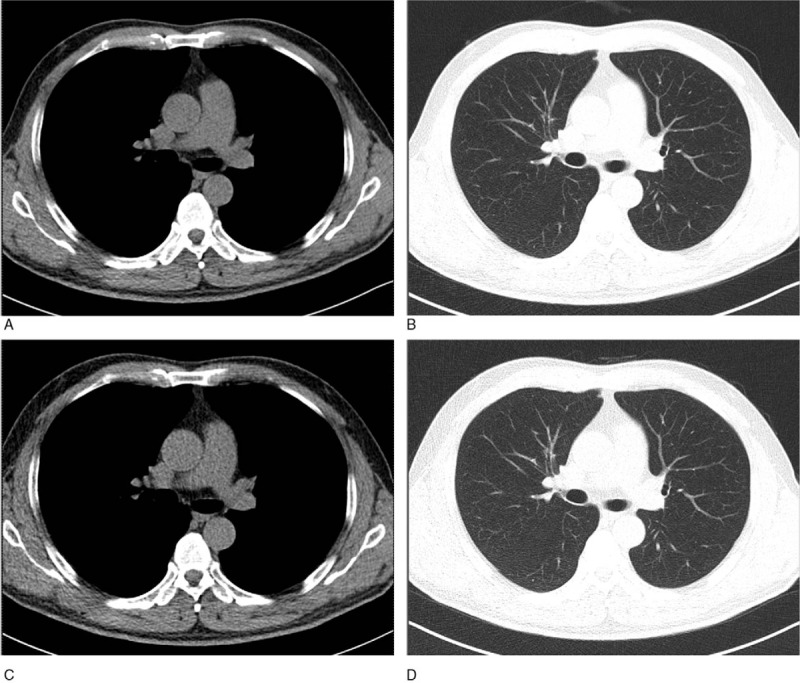
Transverse unenhanced multidetector images obtained with AP scout view (A and B) and AP-lateral scout view (C and D) shows roughly equal image quality on mediastinal (A and C) and lung (B and D) windows CT images, respectively. AP = anteroposterior, CT = computed tomography.

The image quality of almost all scans was rated as excellent or good by both radiologists (Fig. 6). Table 4 shows there was no significant difference in the mean image quality scores of scans acquired after the AP-lateral topogram compared with scans acquired after the AP topogram (*P* = .65/.71).

**Table 4:**
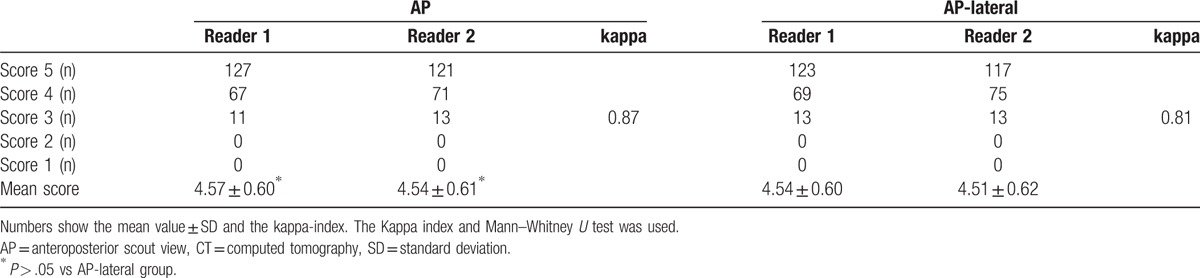
Subjective image quality scores of chest CT examinations in 410 patients.

### 3.4 Reproducibility analysis

For subjective image quality assessment, the correlation coefficients for interobserver agreement in the test and the control groups were 0.81 (95% CI 0.73 to 0.88) and 0.87 (95% CI 0.82 to 0.91), respectively. The correlation coefficients for intraobserver agreement were 0.87 (95% CI 0.73 to 0.88) and 0.88 (95% CI 0.83 to 0.92), respectively (Table 4).

## 4 Discussion

In our prospective study involving a chest phantom and patients who underwent routine thoracic CT examinations at our hospital, we found that an AP scout combined with an additional lateral scout view using the Care Dose 4D significantly reduced the radiation dose without compromising image quality. The study also revealed that when an additional lateral scout view is used, the radiation dose at different off-center positions remained essentially constant.

The phantom study revealed that radiation dose decreased by approximately 50% at the isocenter when an AP-lateral scout view was used. As the table height changed, radiation doses increased by 34% at the highest table position (closest to the x-ray tube) and decreased by 32% at the lowest table position associated with AP scout view. These results support the findings of previous studies.^[11,18,25]^

The ATCM implemented by Siemens (Care Dose 4D) automatically modulates the tube current in relation to patient size and attenuation characteristics together with real-time angular dose modulation during the scan. The technique uses scout views to estimate patient size and automatically adapts radiation dose by matching the actual patient to a reference patient. The tube current will be reduced for smaller patients and increased for larger patients.

The aforementioned changes in radiation dose occurred possibly because moving the phantom away from the gantry center in the vertical direction results in magnification or reduction of scout views.^[18,26]^ Consequently, inappropriate patient centering may overestimate or underestimate patient habitus, thereby causing a change in radiation dose. However, the radiation dose on different off-center positions was essentially unchanged when an AP-lateral topogram was used. According to the manufacturer, Care Dose 4D evaluates the topogram for attenuation in the AP and lateral directions, and calculates the appropriate axial tube current profiles. Using a single AP scout view, the attenuation information from only 1 direction is obtained, and the attenuation in the perpendicular direction is estimated sophistically. By using an AP-lateral topogram, Care Dose 4D can more accurately measure the attenuation and the geometrical width along the patient's long axis.^[16]^ Accordingly, using an AP-lateral topogram avoids misestimating the patient's habitus to some extent.

In terms of the ALARA principle, it has always been a hotspot in CT research to reduce the radiation dose. Lowering tube potential is the most simple and direct method,^[12]^ but too low of a tube potential may increase noise and change the tissue contrast. In 1997, Hopper et al^[27]^ proposed that incremental breast shields could substantially reduce dose. But researchers noted that shields will generate more image noise, artificially augment attenuation values and produce streak artifacts.^[28]^ Furthermore, organ-based tube current modulation techniques can yield nearly the same amount of dose reduction to the breast without increasing image noise.^[29]^ Iterative reconstruction is a postprocessing technique that can lower dose significantly while maintaining image quality.^[14,30]^ However, blotchy and pixilated artifacts have been observed on some images.

ATCM systems are now commonly used to reduce radiation exposure.^[31,32]^ Our finding that radiation dose decreased by 50% when an AP-lateral scout view was used is similar to the results of Rodrigues et al,^[33]^ who combined a routine AP scan with an additional lateral scout view in CT pulmonary angiography. Their findings indicated that an additional lateral topogram could reduce scan length and thereby significantly reduce organ dose. In a cadaver study, Singh et al^[15]^ recently also found that, compared with using an AP scout view alone, incorporating an additional lateral scout view significantly reduced radiation dose in thoracic and abdominal CT. Unfortunately, these authors did not compare image quality. Radiation dose reduction frequently is accompanied by loss of image quality due to increasing noise. In our study, the mean objective quality score was significantly decreased on an AP-lateral scout view compared with using an AP scout view alone. Nevertheless, qualitative image quality was rated as equal. Therefore, the differences in objective image quality seem to be inappreciable for clinical diagnosis.

In the analysis of patients who had received a CT scan in the previous year using a single AP scout view, we found a 45.5% reduction in the CTDIvol and up to a 78.9% reduction in the maximum dose. The dose reduction was only 12.4% in 1 patients and that could potentially be linked to that his weight gain exactly equal to 2 kg between 2 medical examinations.

Other than the different scout views having an impact on the radiation exposure, other factors may have affected our results. The 2 groups did not differ with regard to baseline patient characteristics or with regard to scan parameters; however, scan length did differ between the 2 groups, and scan length has a direct influence on DLP. In patient scans planned on AP-lateral scout views, scan lengths were significantly shorter than those planned on AP-only scout views. This might be because the lateral topogram can display the base of the lungs more clearly.^[33]^ When the AP topogram was used alone, it was sometimes difficult to identify the base of the lungs. In the phantom study, the differences in DLP between the 2 protocols were not due to scan length because scan length was held constant.

This study had several limitations. First, our study included only healthy individuals who underwent medical examination at our hospital, so we did not compare the capacity of the 2 different protocols to discriminate lesions from surrounding normal tissue. Secondly, we restricted our study to evaluating the effect of an additional lateral topogram on radiation exposure and image quality during a nonenhanced scan. Hence, the reliability of multiphase CT scanning and the ability to diagnose lesions needs to further investigated. Thirdly, we only used ATCM techniques in this study. The combined use of ATCM and automated tube voltage selection might allow further reduction of radiation exposure while maintaining good image quality. We plan to conduct additional studies to determine the impact of automated tube voltage selection on reducing radiation exposure.

## 5 Conclusion

In conclusion, our study shows that by combining a routine AP scout view with an additional lateral view using the Care Dose 4D, radiation dose can be significantly reduced without impacting image quality. Hence, using an AP-lateral topogram is a simple and feasible method to achieve lower radiation doses during chest CT screening.

## Acknowledgments

The authors acknowledge Jin Pu and Yuming Li for their great support.
